# Variations of circulating miRNA in paediatric patients with Heart Failure supported with Ventricular Assist Device: a pilot study

**DOI:** 10.1038/s41598-020-62757-7

**Published:** 2020-04-03

**Authors:** Rosetta Ragusa, Arianna Di Molfetta, Romina D’Aurizio, Serena Del Turco, Manuela Cabiati, Silvia Del Ry, Giuseppina Basta, Letizia Pitto, Antonio Amodeo, Maria Giovanna Trivella, Milena Rizzo, Chiara Caselli

**Affiliations:** 1 Institute of Life Sciences, Scuola Superiore Sant’Anna, Pisa, Italy; 2 Institute of Clinical Physiology, CNR, Pisa, Italy; 3 Department of Cardiothoracic Surgery, Ospedale Pediatrico Bambino Gesù, Rome, Italy; 4 Institute of Informatics and Telematics, CNR, Pisa, Italy

**Keywords:** miRNAs, Cardiac device therapy

## Abstract

Circulating miRNAs (c-miRNAs) are promising biomarkers for HF diagnosis and prognosis. There are no studies on HF pediatric patients undergoing VAD-implantation. Aims of this study were: to examine the c-miRNAs profile in HF children; to evaluate the effects of VAD on c-miRNAs levels; to *in vitro* validate putative c-miRNA targets. c-miRNA profile was determined in serum of HF children by NGS before and one month after VAD-implant. The c-miRNA differentially expressed were analyzed by real time-PCR, before and at 4 hrs,1,3,7,14,30 days after VAD-implant. A miRNA mimic transfection study in HepG2 cells was performed to validate putative miRNA targets selected through miRWalk database. Thirteen c-miRNAs were modified at 30 days after VAD-implant compared to pre-VAD at NSG, and, among them, six c-miRNAs were confirmed by Real-TimePCR. Putative targets of the validated c-miRNAs are involved in the hemostatic process. The *in vitro* study confirmed a down-regulatory effect of hsa-miR-409-3p towards coagulation factor 7 (F7) and F2. Of note, all patients had thrombotic events requiring pump change. In conclusion, in HF children, the level of six c-miRNAs involved in the regulation of hemostatic events changed after 30 days of VAD-treatment. In particular, the lowering of c-miR-409-3p regulating both F7 and F2 could reflect a pro-thrombotic state after VAD-implant.

## Introduction

Paediatric heart failure (HF) is a condition at high social economic impact, depending on the frequent need for surgical procedure and the significant morbidity and mortality. It was estimated that among 11000 and 14000 children were annually hospitalized for HF^1^. Recent data indicated that congenital heart disease (CHD) and dilated cardiomyopathy (DCM) are the most common reason for HF and heart transplantation in paediatric patients^2^.

Given the significant morbidity and mortality of HF patients and the gap between availability and demand of donor organ, the use of alternative strategy to transplant have become crucial for the management of end-stage HF patients. In the recent years, the use of Ventricular Assist Device (VAD) for the treatment of end-stage HF children as bridge to cardiac transplantation was increased and the impact of VAD-depended heart unloading increased the survival waitlist and improved the post-transplant outcome^3,4^. However, coagulation disorders, infection, neurological events and device malfunction were described as the common adverse events affecting the children supported with VAD^5^.

Recent studies using next-generation sequencing technologies for comprehensive cardiac coding and non coding RNA expression profiling revealed distinct relative abundance, expression pattern, and genomic origin of different RNA species in human heart, highlighting the different biological roles of the individual RNA classes during evolution^6,7^. In particular, microRNAs (miRNAs) are a family of single-strand, non-coding, small RNAs (~22 nucleotides) that have emerged as important regulators of gene expression in a sequence-specific manner at post-transcriptional level^7^. Changes in the expression of specific miRNAs and the re-expression of foetal miRNAs have been found involved in many of the aberrant processes that characterize myocardial disease in adult patients^8–10^. It has been clear from the beginning that these molecules are remarkably stable in the blood, even under several extreme conditions^11,12^, thus increasing the number of studies investigating the potential role of circulating miRNAs (c-miRNA) as biomarkers, also for HF^13^.

To date, a panel of c-miRNA differentially expressed in HF adult patients compared to healthy control was identified^14^. Furthermore, differential levels of several c-miRNAs able to discriminate HF adult patients with preserved ejection fraction (HFpEF) compared to HF patients with reduced ejection fraction (HFrEF) were observed^13,15,16^. Likewise, the prognostic role of c-miRNAs was investigated and preliminary data showed their potential role in predicting response to VAD therapy for miR-1202^17^, miR-499 and miR-208a/208b^18^, and miR-30e-5p^19^. Few studies exploring the role of c-miRNAs in blood samples of paediatric patients are present^20–22^.

Thus, given the limited information about the role of c-miRNAs in HF children supported by VAD, the aim of this study was to evaluate the c-miRNA profile in serum samples from paediatric patients with HF submitted to VAD implant. Specifically, the levels of c-miRNAs were determined in paediatric patients with HF during time course up to 1 month after VAD treatment; moreover, potential targets of the selected c-miRNAs were identified by an *in silico* analysis, and the regulatory role of selected c-miRNAs on predicted targets was validated by a dedicated *in vitro* study.

## Results

**Figure 1:**
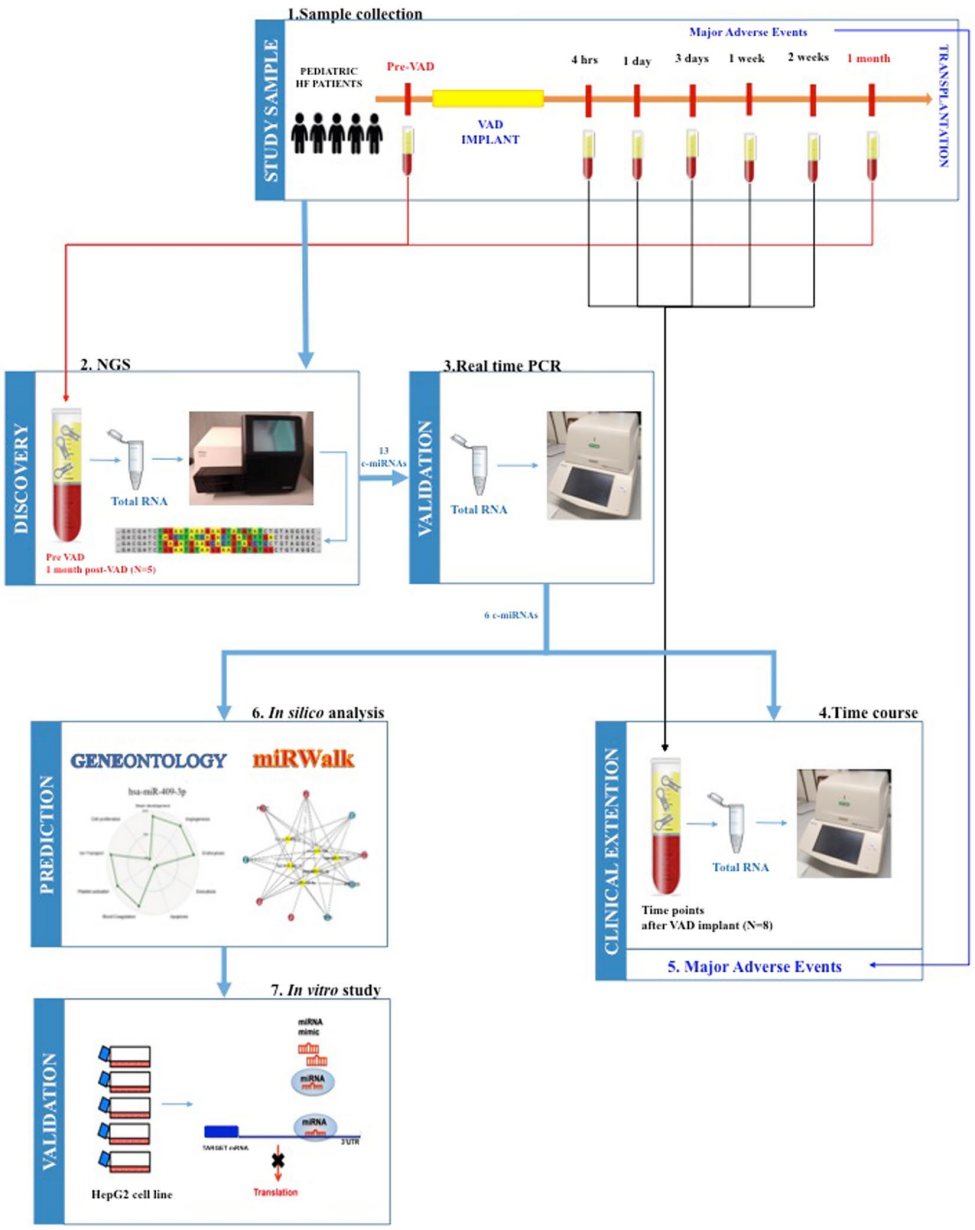
Experimental design. In clinical setting, serum samples were collected from HF children (n = 5, study sample) before and during time course up to 1 month after VAD implant (1). In order to identify those c-miRNAs that were modified by VAD therapy, RNA was isolated from serum samples before and after 1 month of VAD treatment, c-miRNA profile was performed by Next Generation Sequencing (NGS) (2, discovery phase), and, after a refined statistical analysis, sequencing results were confirmed by Real-Time PCR (3, validation phase). Results were clinically extended and the previously identified c-miRNAs were evaluated by Real-Time PCR in serum samples collected at different time points during time course up to 1 month after VAD implant (4) and associated with patients adverse events occurred during time course after VAD implant up to transplantation (5). Finally, to order to predict the putative targets of the selected c-miRNAs, an *in silico* analysis was performed using Gene Ontology and miRWalk software (6) and the identified and clinically selected targets were validated by an *in vitro* transfection study in HepG2 cell line using specific miRNA-mimics (7).

Results from this study were reported following the experimental design of the study detailed in Fig. 1.

### Clinical characteristics of paediatric patients with HF

Pre-procedural features and post-operative clinical data (1 moth after VAD implant) of HF children were reviewed in Table 1. The median age was 25.25 ± 10.9 months and 3 subjects were males. The mean value of LVEF% was 21.7 ± 5.6 and all patients showed an Interagency Registry for Mechanically Assisted Circulatory Support (INTERMACS) profiles 1–2. Overall, 1 month of VAD therapy determined an improvement of cardiac function, as assessed by echocardiography. Accordingly, a parallel significant decrease of circulating levels of NT-pro brain natriuretic peptides (NT-proBNP) was observed after VAD implant compared to pre-VAD. Coagulation indexes, such as a PTT and INR, clinical blood parameters, and other cardiac biomarkers, such as soluble suppressor of tumorigenicity 2 (sST2) and cardiac troponin I (cTnI), did not changed after 1 month of VAD therapy (Table 1).

**Table 1:** Clinical, imaging and bio-humoral data of study sample before and at 1 month after VAD implant.

	Pre-VAD	Post-VAD (1month)	P-value
Age, months	25.25 ± 10.9	—	
Male gender	3 (8)	—	
Etiology, n (%)			
DCM	75%	—	
RCM	12.5%	—	
LV non compaction	12.5%	—	
Weight (Kg)	9.2 ± 1.96	9.6 ± 1.8	ns
LVEF (%)	21.7 ± 5.6	36.4 ± 5	p = 0.0274
LVEDV (mL)	60.3 ± 6.9	20 ± 1.5	p < 0.0001
LVESV (mL)	45.2 ± 2.5	11.78 ± 1.7	p = 0.0002
LVEDD (mm)	18.37 ± 3.5	35.25 ± 2.7	p = 0.0076
LVESD (mm)	42.75 ± 4.1	31 ± 2.8	p = 0.0434
TAPSE (mm)	1.04 ± 0.094	0.65 ± 0.114	p = 0.0225
RVFAC (%)	35.37 ± 3.5	37.12 ± 4.79	ns
White blood cells	11.23 ± 1.6	14.37 ± 3.13	ns
Hb	12.75 ± 0.5	9.9 ± 0.6	p = 0.005
Platelets	323.14 ± 63.59	408.3 ± 41.7	ns
aPTT	46.8 ± 10.22	111.1 ± 47.9	ns
INR	1.21 ± 0.037	1.35 ± 0.31	ns
Glucose (mg/dL)	112.8 ± 11	106 ± 9.49	ns
NT-proBNP (ng/L)	10888.6 ± 3169.7	4071 ± 1914.4	p = 0.0068
sST2 (ng/mL)	89.99 ± 44.6	56.51 ± 17.7	ns
cTnI (ng/L)	124.4 ± 95.66	97.15 ± 49.48	ns
Urea nitrogen (mg/dL)	23.37 ± 8.5	35.1 ± 13.5	ns
Creatinine (mg/dL)	0.348 ± 0.048	0.439 ± 0.19	ns
Albumin (g/dL)	5.01 ± 0.626	4.12 ± 0.12	ns
C-reactive Protein (mg/dL)	0.619 ± 0.254	3.51 ± 1.63	ns
Bilirubin tot (mg/dL)	1.2 ± 0.358	0.431 ± 0.062	p = 0.0397
Lactate Dehydrogenase (U/L)	730.6 ± 102.22	1257 ± 274.1	ns

The clinical outcome of patients was reported in Table 2. During VAD implant, all patients had thrombotic events of the Berlin Heart Chamber requiring pump change. Three patients had neurological complications, five had infection, and no patients had bleeding. After the period of VAD treatment (179 ± 34.71 days), all patients were submitted to transplantation except for one patient in which recovery of cardiac function occurred after VAD implant (Supplementary Table S2).

**Table 2:** Clinical outcome of patients during VAD treatment.

Patients	Infection	Neurological complications/Stroke	Bleeding	Thrombosis	Time of device replacement for Pump Thrombosis
1*	1	1	0	1	1 month
2*	0	1	0	1	3 months
3*	1	0	0	1	2 changes in 1 month
4*	0	0	0	1	1 month
5*	0	0	0	1	2 months
**6**	1	1	0	1	2 months
**7**	1	0	0	1	1 month
**8**	1	0	0	1	1 month

*Patients used for NGS analysis

### c-miRNAs profiling

**Figure 2:**
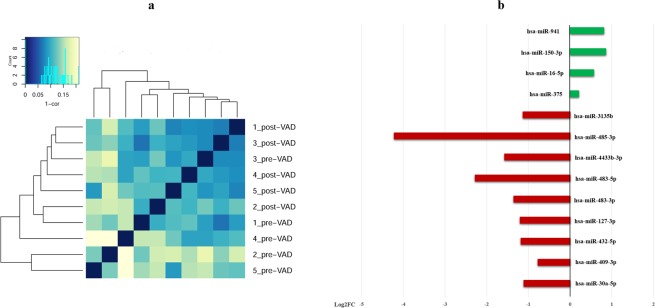
miRNA profiling details. (**a**) Hierarchical clustering of the total c-miRNAs profiles; (**b**) Differentially expressed c-miRNAs from NGS experiments. Log_2_ fold changes between pre- and post-VAD are showed (red bar: downregulated c-miRNAs; green bar: upregulated c-miRNAs).

NGS technology was employed to obtain miRNAs profile in serum samples collected from a group of paediatric patients with HF (N = 5, Supplementary Table S1) at the moment of VAD implant and after 1 month of treatment. Overall, an average of 340 c-miRNAs were identified in each sample and 169 were present in all samples. A hierarchical clustering analysis of the total c-miRNAs profiles (Fig. 2a) showed that, globally, they did not group in pre- and post-VAD samples. Nevertheless, a total of 13 c-miRNAs resulted differentially abundant in serum samples at pre-VAD compared to post-VAD (adj-p < 0.1). Among these, 9 c-miRNAs were downregulated and 4 c-miRNAs upregulated in the post-VAD samples respect to pre-VAD (Fig. 2b). A detailed list of the differentially expressed c-miRNAs is reported in Supplementary Table S3.

### Validation of sequencing data by Real-Time PCR

**Figure 3:**
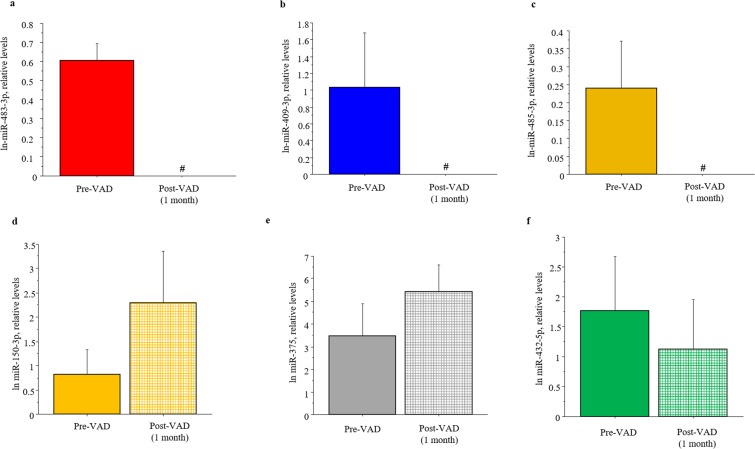
Real-Time PCR data analysis of differentially expressed c-miRNAs. (**a**) hsa-miR-483-3p; (**b**) hsa-miR-409-3p; (**c**) hsa-miR-485-3p; (**d**) hsa-miR-150-3p; (**e**) hsa-miR-375; hsa-miR-432-5p in serum samples of HF children at pre-VAD compared to post-VAD; # Not Detectable.

In order to validate sequencing results, we quantified the 13 c-miRNAs also by Real-Time PCR. Among them, six c-miRNAs showed the same differential expression profile as indicated by NGS experiments. Specifically, hsa-miR-483-3p, hsa-miR-409-3p and hsa-miR-485-3p decreased up to undetectable levels after 1 month of VAD treatment compared to pre-VAD values (Fig. 3a–c). hsa-miR-432-5p showed a trend to decrease, while hsa-miR-150-3p and hsa-miR-375 to increase after VAD implant (Fig. 3d–f). These six miRNAs were selected for the downstream analysis.

### Serum changes of selected c-miRNA during the time-course post-VAD implant

**Figure 4:**
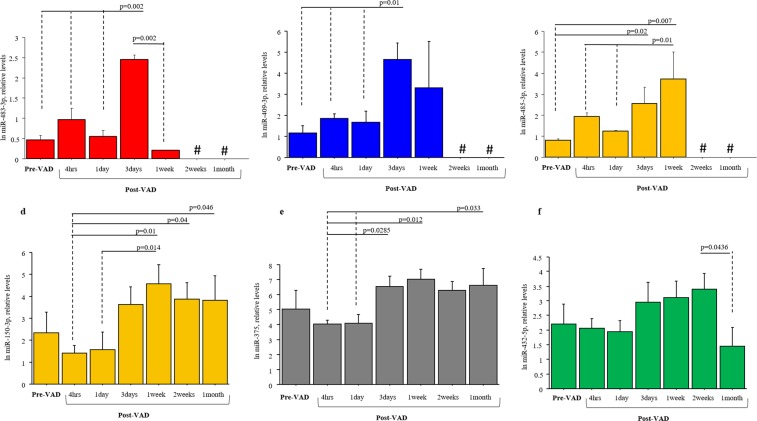
Changes of miRNA levels in the serum samples of HF children during the time-course post-VAD implant. In particular: (**a**) hsa-miR-483-3p; (**b**) hsa-miR-409-3p; (**c**) hsa-miR-485-3p; (**d**) hsa-miR-150-3p; (**e**) hsa-miR-375; hsa-miR-432-5p. Data obtained by Real-Time PCR; # Not Detectable.

In the total group of patients, we evaluated the serum level of the six miRNAs which showed the same trend in NGS and Real-Time PCR analysis at different time points after VAD implant. Serum levels of hsa-483-3p, hsa-409-3p and hsa-miR-485-3p increased up to 3 days but were undetectable after 1 week post-VAD implant (Fig. 4a–c). Conversely, circulating levels of hsa-miR-150-3p, hsa-miR-375, and hsa-miR-432-5p showed a trend to decreased up to 1 day after VAD implant and, gradually increased up to 1 week ant variation during time course (Fig. 4d–f).

### In silico prediction of putative c-miRNAs targets

**Figure 5:**
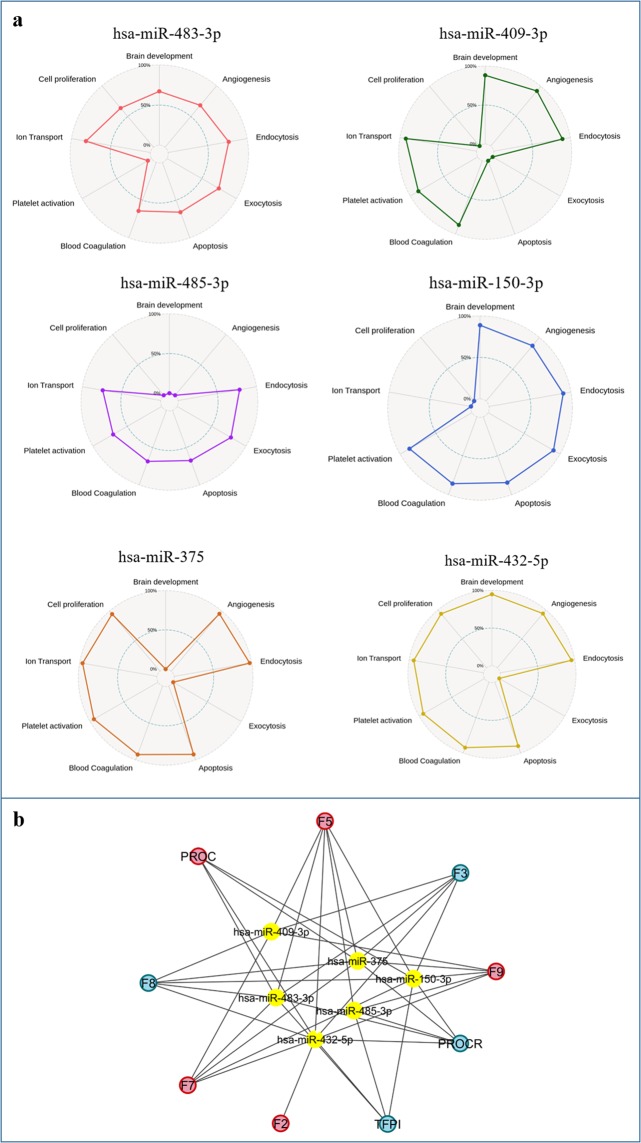
In *silico* analysis. (**a**) Radar charts of the relevant biological processes simultaneously modulated by the selected c-miRNAs after GOBP analysis. The levels show the proportion of the number of putative gene targets for each class. (**b**) Gene target study: c-miRNAs network. The yellow circles represent the six selected c-miRNAs, the blue circles indicate the coagulation factors produced by endothelium, and the red circles show the coagulation factors produced by liver. Arrows indicate the relationship between each c-miRNA and its putative target gene.

To elucidate the potential functions of the selected c-miRNAs, we retrieved from miRWalk2 the putative targets of each six c-miRNAs and searched for the enriched biological process (Gene Ontologies terms) in the c-miRNA-target lists. The analysis showed that a total of 193 biological processes were common to at least 3 miRNAs. Among the clinically relevant biological processes, blood coagulation, platelet activation and membrane trafficking were the main mechanisms simultaneously modulated by the selected c-miRNAs (Fig. 5a). A detailed list of the relevant biological processes simultaneously modulated by the selected c-miRNAs after GOBP analysis is reported in Supplementary Table S4.

Whereas coagulation disorders were reported among the main adverse events for HF children supported by VAD^5^, we decided to investigate the role of the selected six c-miRNAs in haemostasis. Furthermore, we used miRWalk2 database to identify among the putative gene targets of the six c-miRNAs, those involved in the haemostasis process. We found thrombin (F2), tissue factor (F3), coagulation factor V (F5), coagulation factor VII (F7), coagulation factor VIII (F8), coagulation factor IX (F9), protein C [(PROC) inactivator of F5a and F8a], receptor of protein C (PROCR), and tissue factor pathway inhibitor (TFPI) (Fig. 5b). Notably, data showed that many of selected c-miRNAs had the same gene target, indicating for these c-miRNAs a synergic regulatory effect on different coagulation factor (Fig. 5b).

### Experimental validation of the identified putative targets of c-miRNA

Since F2, F5, F7, F8, F9 and PROC were expressed in liver, HepG2 cells were used for the validation of putative targets of selected c-miRNA by miRNA-mimic transfection study.

**Figure 6:**
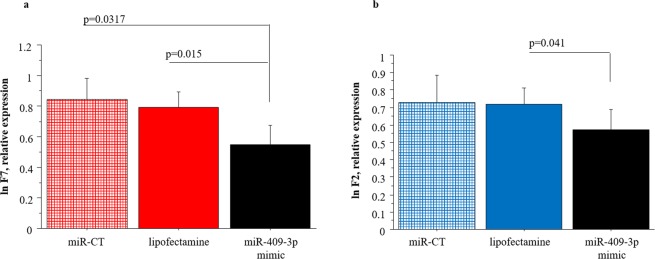
Study transfection results. (**a**) Comparison of F7 expression between HepG2 transfected with miR409-mimic and HepG2 treated with control-miRNA (miR-CT) or lipofectamine; (**b**) Comparison of F2 expression between HepG2 transfected with miR409-mimic and HepG2 treated with control-miRNA (miR-CT) or lipofectamine. Data obtained by Real-Time PCR.

The expression levels of F7 decreased significantly in cells 48 h after transfection with hsa-miR-409-3p mimic compared to cells transfected with control miRNA (miR-CT) mimic or lipofectamine alone (Fig. 6a). Moreover, F2 expression levels significantly decreased after transfection of hsa-miR-409-3p compared with control cells treated only with lipofectamine (Fig. 6b). These results confirmed the regulatory role of hsa-miR-409-3p towards F7 and F2 in the HepG2 *in vitro* model.

The other five selected c-miRNAs had no effect on their putative targets after transfection in HepG2 cells.

## Discussion

In the last few years, the use of VAD has become a feasible and valuable therapeutic approach for HF children. Thus, the careful assessment of molecular mechanisms modified by VAD treatment could be helpful to improve the positive effects of VAD treatment^23,24^. Therefore, for the first time in this pilot study, the profiling of miRNA present in serum samples of HF children during time-course up to 1 month after VAD treatment allowed to identify specific c-miRNAs underling thrombotic process, that, as a matter of fact, occurred in all patients. Specifically, we found that:

- c-miRNAs levels were modified in HF children during the time-course after VAD implant, as assessed by NGS and Real-Time PCR;
- the selected c-miRNAs were involved in regulation of factors affecting haemostasis, as indicated by in silico analysis and confirmed by the dedicated *in vitro* study.
- all HF children had thrombotic events of the Berlin Heart Chamber requiring pump change, clinically confirming the presence of a thrombotic phenotype in these patients after VAD implant.

Modifications of the normal haemostasis by VAD implant has been well documented^25,26^. The blood exposure to the titanium alloy surface of pumps, and the polyester inflow and outflow grafts cause a quickly but moderated activation of coagulation factor XII followed by platelet, leukocytes and complement activation^24^. At the same time, the shear force induces the activation of coagulation by endothelium, platelets and leukocytes stimulation^26^. Hence, a correct antithrombotic therapy and monitoring strategy are vital for these patients^27^. Currently, the most common anticoagulant agents used for children treatment are those used in adults and included unfractionated heparin, vitamin K antagonist, warfarin, and low molecular weight heparin. Optimal dosing of these drugs is not clear for children and no randomized clinical trials aimed at determining safety and efficacy of therapy have been completed^24^. On the whole, data from this study could suggest a potential role for c-miRNAs, in particular for hsa-miR-409-3p as early biomarker for the monitoring of adverse thrombotic event in paediatric patients supported with VAD, especially taking into account that all of the studied patients had a thrombotic events during the first three months after VAD implant causing the pump replacement. Moreover, considering that the classical coagulation indexes were not modified after 1 month of VAD therapy and that the optimal intensity of antithrombotic therapy is unknown, having identified few miRNAs as molecules involved in the regulation of coagulation during time-course after VAD implant could allow their use to monitor pharmacological therapy and prevent the occurrence of related adverse events.

Among all selected miRNAs, a regulatory activity on haemostasis was previously reported only for hsa-miR-409-3p. Fort *et al*. found that the hsa-miR-409-3p directly targets the 3′-UTR of transcript variant β of fibrinogen and that the overexpression of this miRNA reduces the fibrinogen production^28^. In another study, Liu *et al*. evaluated in adult patients with atrial fibrillation changes of c-miRNAs levels after catheter ablation compared to baseline values^29^. Indeed, in these patients at high risk of thrombosis, the blood flow congestion in the atrium and the irregular atrial wall motion abnormalities could increase the possibility of coagulation activation^29^. Liu *et al*. found that c-miR-409-3p levels were lower in atrial fibrillation patients at the baseline when compared with healthy subjects, suggesting that this condition could promote an increase of fibrinogen levels and consequently an increased risk of thrombosis. Conversely, after catheter ablation, the circulating levels of hsa-miR-409-3p were comparable to the levels of miRNA of the control group^29^. Data from Liu *et al*. could be in agreement with those from our study where levels of c-miRNA-409-3p were undetectable in serum from HF children already after 3 days post-VAD, thus suggesting an increased risk of thrombosis.

Moreover, it has been observed that miRNA-16, increased after VAD implant by NGS analysis but not confirmed by Real-Time PCR in our study, exerted a negative effect on endothelial repair through the inhibition of nitric oxide (NO) production in an experimental animal model^30^. An intriguing hypothesis might be proposed in light of these results, possibly attributing a haemostatic regulatory role to miR-16, whose circulating levels increased in HF children after VAD implant in our study. It is possible to speculate that an increasing in miR-16 could lead to a reduction of NO bioavailability, that in turn, could overcome its protective anti-aggregation action^31^, resulting in uncontrolled balance between pro- and anti-aggregants, as observed in this study. Very importantly, a systemic antagonizzation of miR-16 was able to prevent its negative effects in an experimental animal model^30^, thus opening to a new targeted therapeutic option.

## Limitation

Due to the low number of the study samples, this work was a pilot study and a real clinical validation could not be well performed. Hence, our results are mainly a proof of concept and further studies are needed to evaluate whether our hypothesis holds true in a large population of pediatric patients submitted to VAD therapy.

## Conclusion

In summary, the present pilot study could suggest that c-miRNAs whose levels were modified by VAD use might be early biomarkers of altered haemostasis in HF children after VAD implant. From a clinical viewpoint, since coagulation disorders are among the major adverse events for HF paediatric patients supported by VAD, the possibility of using an early biomarker of thrombosis in this population would allow an early identification of haemostatic dysregulation and, as a consequence, an appropriately adaptation of the pharmacological therapy, thus improving the outcome of patients.

Further studies performed in larger and more homogeneous population are needed to better evaluate the clinical relevance of c-miRNAs as biomarkers of thrombosis and if their use could improve diagnosis and prognosis for children with HF in the current clinical setting, also addressing for a personalized management and a targeted therapy for HF paediatric patients.

## Materials and Methods

### Experimental design and study groups

The experimental design of this study, reported in Fig. 1, included the following steps:

1. serum samples were collected from HF children before and during time-course up to 1 month after VAD implant.
2. c-miRNAs profile was performed by *Next Generation Sequencing* (NGS) using RNA isolated from serum samples before and after 1 month of VAD treatment.
3. sequencing results were confirmed by Real-Time PCR and c-miRNAs whose trend of modulation were concordant in NGS and Real-Time PCR analysis were selected for further analysis.
4. Real-Time PCR were performed using RNA isolated from serum sample collected at different time points during time-course up to 1 month after VAD implant.
5. the relationship with major adverse events occurred in patients during time course after VAD implant up to transplantation was studied.
6. an *in silico* analysis was performed to discover the putative targets of selected c-miRNAs.
7. an *in vitro* transfection study using miRNA-mimics in HepG2 cell line was carried out for testing the regulatory role of selected c-miRNAs on predicted targets.

A total of eight pediatric patients undergoing VAD implantation at the Cardiovascular Department of Ospedale Bambino Gesù of Rome from 2013 to 2015 were enrolled in the study. NGS analysis and sequencing validation by Real-Time PCR were carried out using a group of five patients (Supplementary material Table S1). Modifications of c-miRNAs levels were evaluated in in the total group of HF children during the time-course after VAD implant by Real-Time PCR.

### Study sample

A total of eight pediatric patients undergoing VAD implantation at the Cardiovascular Department of Ospedale Bambino Gesù of Rome from 2013 to 2015 were enrolled in the study. All paediatric patients were implanted with pulsatile-flow pump (Supplementary Table S2). Clinical, biohumoral and echocardiographic data were obtained at VAD implant (pre-VAD) and at 1 month after implantation. The clinical course of these patients was assessed considering major adverse events after VAD implant, including infection, neurological complication/stroke, bleeding, and thrombosis. The serum samples of paediatric patients were collected through the peripheral vein before VAD implant and during the time course at 4 hours, 1 day, 3 days, 7 days, 14 days up to 30 days after surgery. The samples were immediately separated by centrifugation for 15 min at 1500xg and aliquots of serum were stored at −80 °C prior to analysis.

This study complied with the principles of the Declaration of Helsinki. Informed consent was given by all parents of children enrolled in this study and the protocol was approved by the Ospedale Bambino Gesù ethic committee.

### RNA extraction from serum

For NGS analysis, total RNA (including small RNA fraction) was isolated from about 1 mL serum using QIAamp circulating nucleic acid kit (Qiagen S.p.a, Milano, Italy) according to manufacturer’s instruction. For Real-Time PCR total serum RNA was isolated from 200 µL serum by RNeasy mini Kit (Qiagen S.p.a, Milano, Italy) as previously described^32^. The total RNA was stored to −80 °C until use.

### Construction of small RNA cDNA libraries and NGS

The small cDNA libraries construction was performed with TruSeq small RNA sample preparation Kit (Illumina) from 50-100 ng of total RNA extracted from serum samples obtained from N = 5 patients at pre-VAD and at 1 month after VAD implant. Clinical characteristics of this sample group was detailed in Supplementary Table S1. The cDNA libraries were loaded at six-plex level of multiplexing into a flow cell V3 and sequenced in a single-reads mode (50 bp) on a MiSeq sequencer (Illumina, San Diego, CA, USA). Raw data were analysed and miRNA identification were performed as described^33^.

### Bioinformatic analysis of identified miRNAs

miRNAs count matrices were analysed using R Bioconductor’s package DESeq2^34^. Hierarchical clustering of samples was performed on regularized log transformed counts (rlog function) using hclust function with Spearman correlation coefficient as distance metric and complete as agglomeration method. To test for the effect of VAD controlling for patient, we applied the likelihood ratio test (LRT) which determined the p-value by the difference in deviance between full (~time + patient) and reduced model formula (~patient). c-miRNAs with Benjamini & Hochberg (1995) adj-p value < 0.1 were selected for downstream validation step using Real-Time PCR.

### Real-Time PCR from serum RNA

A validation study by Real-Time PCR was carried out using the group of five patients used for NGS analysis to confirm sequencing data. Moreover, Real-Time PCR was also performed using RNA isolated from serum samples of the total group of eight patients collected at different time points during time course up to 1 month after VAD implant.

The reverse transcription of c-miRNA was performed from 10 µL of RNA extracted from 200 µL serum using *miScript II RT Kit* (Qiagen S.p.a, Milano, Italy) according to the manufacturer instruction.

Real-Time PCR reactions were performed in duplicate in the CFX-96 Real-Time PCR detection systems (Bio-Rad) using SsoFAST EvaGreen Supermix (Biorad, Hercules, CA, USA). Amplification of cDNA, obtained from c-miRNA, was carried out using primer pairs formed by the miScript Universal primer (Qiagen S.p.a, Milano, Italy) and a primer having the same sequence of the c-miRNA to be analysed (Supplementary Table S5). The reaction mixture included 5 μL of cDNA template (1:50), 0.75 μL of each primer (Sigma-Aldrich, St. Louis, MI, USA), 10 μL SsoFAST EvaGreen Supermix, and 3.5 μL sterile H_2_O for each reaction. Normalization of c-miRNAs levels was performed using the exogenous reference gene *cel*-miR-39.

### Prediction of miRNA Targets

Predicted c-miRNAs-gene targets were selected using miRWalk 2.0 database (http://zmf.umm.uni-heidelberg.de/apps/zmf/mirwalk2), which provides the largest available collection of miRNA-target interactions obtained from 12 established prediction programs. For each list of putative targets, the Gene Ontology (GO) analysis was performed to identify the enriched biological processes (BP) in which c-miRNAs could be involved. An adjusted (adj)-p value < 0.05 was considered statistically significant.

### Cell culture

The HepG2 cell line were grow in 75 cm^2^ flasks using EMEM media (Invitrogen, Carlsbad, CA, USA), supplemented with 10% FCS, 50 U/mL penicillin, 50 μg/mL Streptomycin, 200 μM L-Glutamine, 10 μg/mL Ciprobay and 2 μg/mL Tetracycline.

### Transfection of HepG2 with miRNA mimics

A transfection study using with miRNA mimics was performed in order to verify the regulatory effect of c-miRNA on the *in silico*-predicted mRNA targets. HepG2 cells (2 × 10^5^) were seeded in 6-wells plates 24 h prior to transfection. 80 nM of synthetic miRNA mimics (Supplementary Table S6) (Shanghai GenePharma, Shanghai, China) were transfected into cells using Lipofectamine 2000 (Invitrogen, Carlsbad, CA, USA). After 6 h of transfection, the medium was replaced with EMEM and, 48 h later, the HepG2 cells were washed twice with PBS and used for total RNA extraction.

### Purification of RNA from cell culture

Total RNA (including small RNA fraction) was extracted from HepG2 cell using miRNeasy mini kit (Qiagen S.p.a, Milano, Italy) according to manufacturer’s recommendations.

The concentration and purity of isolated total RNA, obtained from cell culture were determined spectrophotometrically through the use of NanoDrop (Thermo Fisher Scientific, Waltham, MA, USA). The total RNA was stored to −80 °C until use.

### Real-Time PCR from cellular RNA

The reverse transcription was performed on 1 μg of total RNA obtained from HepG2 cells using *IScript cDNA Synthesis Kit* (Bio-Rad Laboratories, Hercules, CA, USA) according to manufacturer’s recommendations. The expression of miRNA putative target genes (Supplementary Table S7) was analyzed in HepG2 by CFX-96 Real-Time PCR detection systems (Bio-Rad). The reaction mixture included 2 μL cDNA template (1:10 diluition), 0.4 μL of each primer (Sigma-Aldrich), 10 μL SsoFAST EvaGreen Supermix; and 7.2 μL sterile H_2_O. Primers for candidate reference genes and coagulation factors were designed using Primer Express (Version 2.0 Applied Biosystems). Normalization of coagulation factors mRNA was carried out using ribosomal protein L13a (RPL13a), peptidylpropyl isomerase A (cyclophillin A) (PPIA) and eukaryotic translation elongation factor 1 alpha 1 (eEF1A) as reference genes.

### Statistical analysis

Quantitative data are presented as mean ± standard error. Not normally distributed data were log transformed. Comparison between pre-VAD vs. 30 days post-VAD was performed by paired t-student test. Differences among groups was compared by one-way and two-way ANOVA followed by Fisher’s post-hoc test. A P-value ≤ 0.05 was considered significant.

## Supplementary information

Supplementary Materials.

## Data Availability

The data that support the findings of this study are available from OPBG and IFC-CNR but restrictions apply to the availability of these data, which were under authorization for the current study and in compliance with GDPR 2016/679, and so are not publicly available. Data are however available from the corresponding author on reasonable request and with permission of OPBG and IFC-CNR.
