# Supplementary material for: Variations of circulating miRNA in paediatric patients with Heart Failure supported with Ventricular Assist Device: a pilot study
Source: Sci Rep. 2020 Apr 3;10:5905. doi: 10.1038/s41598-020-62757-7 (PMC7125126; doi:10.1038/s41598-020-62757-7)
Supplement: Supplementary file 1 — Supplementary Materials. [file 41598_2020_62757_MOESM1_ESM.pdf]

SUPPLEMENTARY MATERIAL

Variations of circulating miRNA in paediatric patients with Heart Failure supported with Ventricular Assist Device: a pilot study.

Rosetta Ragusa, PhD[a,b] Arianna Di Molfetta, MD[c] Romina D'Aurizio, PhD[d] Serena Del Turco, PhD[b] Manuela Cabiati, PhD[b] Silvia Del Ry, MSc[b] Giuseppina Basta, MSc[b] Letizia Pitto, PhD[b] Antonio Amodeo, MD[c] Maria Giovanna Trivella, MD[b] Milena Rizzo, PhD[b#] Chiara Caselli. PhD[b#*]

Table of contents:

1. **Supplementary Table S1**. Clinical characteristics of N=5 patients used for NGS analysis and methodological data validation by real-time PCR Pag. 3
2. **Supplementary Table S2**. VAD treatment data of HF paediatric patients. Pag. 4
3. **Supplementary Table S3.** List of the c-miRNAs differentially regulated after 1 month of VAD treatment. Pag. 5
4. **Supplementary Table S4.** The main physiological mechanisms in which all six c-mirRNAs are involved**.** Pag. 6
5. **Supplementary Table S5.** Primer sequences for miRNAs. Pag. 8
6. **Supplementary Table S6.** miRNA mimic sequences for HepG2 transfection. Pag. 9
7. **Supplementary Table S7.** Primer sequences for coagulation factors and reference genes. Pag. 10

Supplementary Table S1. Clinical characteristics of N=5 patients used for NGS analysis and methodological data validation by real-time PCR**.**

	**Pre-VAD**	**Post-VAD (1month)**	**P-value**
**Age, months**	13.8±6.25	-	
**Male gender**	2 (5)	-	
**Etiology, n (%)**			
**DCM**	80%	-	
**LV non compaction**	20%	-	
**Weight (Kg)**	6.2±0.9	7.02±0.919	ns
**LVEF (%)**	16.6±1.7	36.9±3.87	p=0.0085
**LVEDV (mL)**	57.8±7.038	20.16±1.6	p=0.004
**LVESV (mL)**	48.4±6.26	11.96±1.73	p=0.0056
**LVEDD (mm)**	48±3.7	39±3.11	ns
**LVESD (mm)**	43.8±3.12	35±3.32	ns
**TAPSE (mm)**	1.15±0.07	0.75±0.15	ns
**RVFAC (%)**	38±3.3	43±5.14	ns
**White blood cells**	10.54±1.47	10.1±1.41	ns
**Hb**	12.7±0.73	9.74±0.83	ns
**Platelets**	339.4±90.87	395.4±47.6	ns
**aPTT**	53.18±13.54	58.16±6.96	ns
**INR**	1.18±0.04	1.48±0.43	ns
**Glucose (mg/dL)**	96.75±12.88	99.2±11.1	ns
**NT-proBNP (ng/L)**	13016.2±5008.9	1848±485.3	p=0.0015
**sST2 (ng/mL)**	119±70.65	28.8±7.6	ns
**cTnI (ng/L)**	38.12±12.1	45.7±21.3	ns
**Urea nitrogen (mg/dL)**	30.2±7.19	15±5.87	ns
**Creatinine (mg/dL)**	0.34±0.07	0.17±0.03	ns
**Albumin (g/dL)**	4.5±0.34	4±0.12	ns
**C-reactive Protein (mg/dL)**	0.6±0.3	1.14±0.37	ns
**Bilirubin tot (mg/dL)**	0.94±0.34	0.39±0.08	ns
**Lactate Dehydrogenase (U/L)**	718.25±138.32	974.5±121.9	ns

Supplementary Table S2. VAD treatment data of HF paediatric patients.

Patient	Diagnosis	Weight (kg)	LVEF% (pre-VAD)	VAD type		VAD duration (day)	Outcome#
**1***	Dilated	5	10	LVAD	*Thoratec, Berlin Heart Excor*	120	Transplant
**2***	LV non compaction	4.7	17	LVAD	*Thoratec, Berlin Heart Excor*	150	Transplant
**3***	Dilated	7.7	17	LVAD	*Thoratec, Berlin Heart Excor*	270	Transplant
**4***	Dilated	9	20	LVAD	*Thoratec, Berlin Heart Excor*	315	Transplant
**5***	Dilated	4.6	19	LVAD	*Thoratec, Berlin Heart Excor*	90	Transplant
**6**	Dilated	15.2	16	LVAD	*Thoratec, Berlin Heart Excor*	296	Recovery
**7**	Dilated	7.7	15	LVAD	*Thoratec, Berlin Heart Excor*	116	Transplant
**8**	Dilated	20	60	BIVAD	*Thoratec, Berlin Heart Excor*	75	Transplant

*Patients used for NGS analysis

#Recovery/Transplant/Death

Supplementary Table S3. List of the c-miRNAs differentially regulated after 1 month of VAD treatment.

	baseMean	Log2FC	p-value	p-adj
**hsa-miR-30a-5p**	319.05	-1.11	4.06E-05	1.99E-03
**hsa-miR-409-3p**	198.63	-0.77	4.90E-04	1.60E-02
**hsa-miR-432-5p**	41.8	-1.18	5.62E-03	8.47E-02
**hsa-miR-127-3p**	97.56	-1.20	2.25E-06	1.47E-04
**hsa-miR-483-3p**	76.98	-1.35	4.96E-20	9.72E-18
**hsa-miR-483-5p**	24.34	-2.28	2.87E-12	2.82E-10
**hsa-miR-4433b-3p**	6.35	-1.57	2.51E-03	6.31E-02
**hsa-miR-485-3p**	4.45	-4.22	4.42E-03	7.88E-02
**hsa-miR-3135b**	15.20	-1.13	5.28E-03	8.47E-02
**hsa-miR-375**	178.43	0.21	8.98E-05	3.52E-03
**hsa-miR-16-5p**	1697.45	0.57	2.89E-03	6.31E-02
**hsa-miR-150-3p**	14.33	0.86	4.07E-03	7.88E-02
**hsa-miR-941**	60.53	0.81	7.03E-03	9.84E-02

baseMean=mean number of reads; Log2FC=log2 Fold change; p-value; p-adj=p-value adjusted ≤0.1

Supplementary Table S4. The main physiological mechanisms in which all six c-mirRNAs are involved**.**

GOBP	c-miRNA involved	GOBP-Fg	GOBP-Bg	BH	Fisher
**Brain development**	**hsa-miR-409-3p**	134	152	0.0435	0.0252
	**hsa-miR-483-3p**	102	152	0.0273	0.0112
	**hsa-miR-150-3p**	135	152	0.0088	0.0015
	**hsa-miR-432-5p**	144	152	0.0123	0.0031
**Angiogenesis**	**hsa-miR-409-3p**	177	192	0.0006	4.1e-05
	**hsa-miR-483-3p**	131	192	0.0103	0.0018
	**hsa-miR-150-3p**	167	192	0.0160	0.0040
	**hsa-miR-432-5p**	180	192	0.0147	0.0045
	**hsa-miR-375**	183	192	0.0101	0.0027
**Blood Coagulation**	**hsa-miR-409-3p**	403	460	0.0041	0.0006
	**hsa-miR-483-3p**	296	460	0.0109	0.0021
	**hsa-miR-150-3p**	395	460	0.0018	0.0002
	**hsa-miR-432-5p**	425	460	0.0053	0.0008
	**hsa-miR-375**	427	460	0.0207	0.0074
	**hsa-miR-485-3p**	315	460	0.0121	0.0016
**Platelet activation**	**hsa-miR-409-3p**	182	207	0.0302	0.0128
	**hsa-miR-483-3p**	11	207	0.0284	0.0135
	**hsa-miR-150-3p**	180	207	0.0131	0.0030
	**hsa-miR-432-5p**	194	207	0.0127	0.0034
	**hsa-miR-375**	195	207	0.0253	0.0115
	**hsa-miR-485-3p**	146	207	0.0243	0.0053
**Ion transport**	**hsa-miR-409-3p**	121	131	0.0041	0.0006
	**hsa-miR-483-3p**	107	131	2.62e-07	4.65e-09
	**hsa-miR-432-5p**	123	131	0.03	0.0158
	**hsa-miR-375**	126	131	0.0129	0.0042

	**hsa-miR-485-3p**	97	131	0.0139	0.0021
**Endocytosis**	**hsa-miR-409-3p**	104	116	0.0343	0.0161
	**hsa-miR-483-3p**	89	116	0.0003	1.42e-05
	**hsa-miR-150-3p**	105	116	0.0070	0.0010
	**hsa-miR-432-5p**	111	116	0.0123	0.0031
	**hsa-miR-375**	113	116	0.0060	0.0012
	**hsa-miR-485-3p**	91	116	0.0013	9.39e-05
**Exocytosis**	**hsa-miR-483-3p**	41	55	0.0229	0.0073
	**hsa-miR-150-3p**	50	55	0.0369	0.0187
	**hsa-miR-485-3p**	43	55	0.029	0.0074
**Apoptosis**	**hsa-miR-483-3p**	449	680	0.0001	4.32e-06
	**hsa-miR-150-3p**	575	680	0.0023	0.0003
	**hsa-miR-432-5p**	613	680	0.0453	0.0282
	**hsa-miR-375**	630	680	0.0093	0.0022
	**hsa-miR-485-3p**	458	680	0.0111	0.00131
**Cell proliferation**	**hsa-miR-483-3p**	209	328	0.0316	0.0154
	**hsa-miR-375**	311	328	0.0024	0.0004
	**hsa-miR-432-5p**	306	328	0.0048	0.0007

Gene ontology biological processes (GOBP) analysis; GOBP-Fg: number of genes predicted as putative targets in a given GOBP; GOBP-Bg: number of genes in a given GOBP; $p<0.05$ BH (Benjamini-Hochberg Procedure); $p<0.05$ Fisher.

Supplementary Table S5. Primer sequences for miRNAs.

miRNAs	Sequence	N. GenBank access
**hsa-miR-16-5p**	TAGCAGCACGTAAATATTGGCG	-----
**hsa-miR-30a-5p**	TGTAAACATCCTCGACTGGAAG	LM_378774.1
**hsa-miR-127-3p**	TCGGATCCGTCTGAGCTTGGCT	LM_379064.1
**hsa-miR-150-3p**	CTGGTACAGGCCTGGGGGACAG	LM_380285.1
**hsa-miR-375**	TTTGTTCGTTCGGCTCGCGTGA	NR_029867.1
**hsa-miR-409-3p**	GAATGTTGCTCGGTGAACCCCT	LM_379542.1
**hsa-miR-432-5p**	TCTTGGAGTAGGTCATTGGGTGG	LM_379772.1
**hsa-miR-483-3p**	TCACTCCTCTCCTCCCGTCTT	LM_379707.1
**hsa-miR-483-5p**	AAGACGGGAGGAAAGAAGGGAG	LM_380378.1
**hsa-miR-485-3p**	GTCATACACGGCTCTCCTCTCT	LM_379710.1
**hsa-miR-941**	CACCCGGCTGTGTGCACATGTGC	LM_380529.1
**hsa-miR-3135b**	GGCTGGAGCGAGTGCAGTGGTG	NR_039668.1
**hsa-miR-4433b-3p**	CAGGAGTGGGGGGTGGGACGT	NR_106995.1
***cel*-miR-39**	TCACCGGGTGTAAATCAGCTTG	LM_608101.1
**U6**	CGCAAGGATGACACGCAAATTC	NR_004394

Supplementary Table S6. miRNA mimic sequences for HepG2 transfection.

miRNAs	mimic
**has-miR-150-3p**	**5'-CUG*GUACAGGCCUGGGGGACAG***UU**-3'**<br>UUGUA***CAUGUCCGGACCCCCUGUC***
**has-miR-375**	**5'-UUU*GUUCGUUCGGCUCGCGUGA***UU**-3'**<br>UUAUU***CAAGCAAGCCGAGCGCACU***
**has-miR-409-3p**	**5'-GAA*UGUUGCUCGGUGAACCCCU***UU**-3'**<br>UUCAA***ACAACGAGCCACUUGGGGA***
**has-miR-483-3p**	**5'-UCA*CUCCUCUCCUCCCGUCUU***UU**-3'**<br>UUUAA***GAGGAGGGGAGGGCAGAA***
**has-miR-485-3p**	**5'-GUC*AUACACGGCUCUCCUCUCU***UU-3'<br>CUU***UAUGUGCCGAGAGGAGAGA***
**has-miR-432-5p**	**5'-UCUU*GGAGUAGGUCAUUGGGUGG***UU-3'<br>UUCU***CCUCAUCCAGUAACCCACC***
**miR-CT**	**5'-CUCU*AGGUUAAACUCCUGGUU***-3'<br>UUGUAA***UCCAAUUUGAGGACCAA***

Supplementary Table S7. Primer sequences for coagulation factors and reference genes.

Gene	Sequence		N. GenBank access
**F2**	Forward	CTCGGGAGAGGCAGACTGT	NM_001311257.1
	Reverse	TCCAGGAGCTCTCTTTCGGT	
**F3**	Forward	TGTTCAAATAAGCACTAAGTCAGGA	NM_001178096.1
	Reverse	TAGGAGAAGACCCGTGCCAA	NM_001993.4
**F5**	Forward	GATGACCCTCCATGCCTCAC	NM_000130.4
	Reverse	CCCTCAGTTAGGGTCCCTTTT	
**F7**	Forward	AGTACTGCAGTGACCACACG	NM_000131.4<br>NM_001267554.1<br>NM_001267554.1
	Reverse	GGATATTCAACTGTGGGTGTGC	
**F8**	Forward	GCACAGATTACTGCTTCATCCT	NM_000132.3
	Reverse	TTGGATTATTCACCTGAGGTCTCC	NM_019863.2
**F9**	Forward	TCACTCAAAGCACCCAATCA	NM_000133.3
	Reverse	TCAAAACAACCTGCCAAGGGA	NM_001313913.1
**PROC**	Forward	GTCACCCCGCAGTGAAGTT	NM_000312.3
	Reverse	ATGAGCCGCGGATCTACTTG	
**PROCR**	Forward	CCTCAGATGGCCTCCAAAGAC	NM_006404.4
	Reverse	TGGTACCACACGTGATAGGG	
**TFPI**	Forward	ACATTGCAACAAGAAAAGCCAGA	NM_001032281.3<br>NM_001318941.2<br>NM_001329239.1<br>NM_001329240.1<br>NM_001329241.1<br>NM_006287.5
	Reverse	ATATTGCCCAGGCATCCACC	
**RPL13a**	Forward	CGCCCTACGACAAGAAAAAG	NM_012423
	Reverse	CCGTAGCCTCATGAGCTGTT	
**PPIA**	Forward	CTTGGGCCGCGTCTCCTTCG	NM_021130
	Reverse	TTGGGAACCGTTTGTGTTTGGGGC	
**eEF1A**	Forward	CTTTGGGTCGCTTTGCTGTT	NM_001402
	Reverse	CCGTTCTTCCACCACTGATT	
